# Negative association between resilience and event-related potentials evoked by negative emotion

**DOI:** 10.1038/s41598-018-25555-w

**Published:** 2018-05-08

**Authors:** Dan Chen, Jianhui Wu, Zhuxi Yao, Kaikai Lei, Yuejia Luo, Zhihao Li

**Affiliations:** 1 College of Psychology and Sociology, Shenzhen University, Shenzhen, P. R. China; 2 Shenzhen Key Laboratory of Affective and Social Cognitive Science, Shenzhen University, Shenzhen, P. R. China; 3 Key Laboratory of Behavioral Science, Institute of Psychology, Chinese Academy of Sciences, Beijing, P. R. China; 4 Department of Psychology, University of Chinese Academy of Sciences, Beijing, P. R. China; 5 Donders Institute for Brain, Cognition and Behavior, Department for Cognitive Neuroscience, Radboud University Medical Centre, Nijmegen, The Netherlands

## Abstract

Individuals with low level of resilience are documented to be less capable of regulating negative emotion. To investigate the underlying neurophysiology, the present study examined the relationship between resilience and the late positive potential (LPP) evoked by emotionally negative pictures. Fifty-four participants watched negative and neutral pictures passively while their electroencephalogram (EEG) was recorded to assess LPP. Participants also completed the Connor-Davidson Resilience Scale (CD-RISC) for assessment of their resilience levels. We found that resilience was negatively correlated with the LPP response to negative emotional pictures. Additionally, this negative correlation was mainly driven by optimism, one of the three composite factors that contribute to resilience. Our results showed a neurophysiological correlate for the effect of resilience on negative emotion, and suggested a predictive value of optimism in identifying individuals potentially sensitive to affective interruptions.

## Introduction

Attention allocation is typically biased in the human brain with emotionally negative information often drawing more attention^1^. This bias is thought to serve crucial evolutionarily adaptive functions to avoid harm^2^. However, in situations like coping with stress, a bias toward negative emotional processing is undesirable or even harmful as excessive emotional arousal may usurp attentional resources, thus hampering ongoing processes more directly related to one’s interests/goals. In these situations, emotion regulation is needed for one to maintain an appropriate attentional balance. Since autonomic emotion regulation varies across individuals^3,4^, studies examining associations between this regulative capacity and different personality factors are of research interest for improving identification and protection of individuals sensitive or vulnerable to aversive emotional distraction.

Among different personal characteristics, resilience, defined as a measure of stress coping ability, could be such a key factor associated with the handling of adversity^5^. Evidence shows that resilience can buffer the negative impact of traumatic events^6^, anxiety^7^ and depression^8^; as well as predict treatment responses for PTSD^9^. For instance, a daily diary study noted that participates with high levels of resilience reported less negative emotion in response to daily stressors^10^. A recent behavioral study has also shown a negative association between resilience and the attentional bias to negative emotional stimuli^11^. However, previous studies of resilience and emotion were mostly conducted at the behavioral level by questionnaires. Based on these previous studies, it is still unclear whether resilience is a predictor of physiological brain responses to negative emotion. Additionally, resilience is, in fact, a relatively general measure with composite factors of tenacity (persistence in maintaining value), strength (being strong with setback), and optimism (positive thinking)^5,12^. These factors may contribute differently in autonomic emotion regulation, thus additional and separate assessments are also desirable.

To examine the predictive value of resilience in brain responses to negative emotion, the present study used linear regression models to determine whether questionnaire measures of resilience predicted event-related potentials (ERPs) evoked by emotional stimuli. Specifically, ERPs evoked by negative versus neutral pictures were contrasted to uncover a late positive potential (LPP)^13^, which indicated a negative attentional bias. The LPP is modulated by emotional intensity and considered a physiological measure of autonomic response to emotion^14,15^. With these measurements of resilience and ERPs, we hypothesized that resilience would predict negative emotional responses, and this predictive power may possess different contribution from its composite factors.

## Results

**Figure 1:**
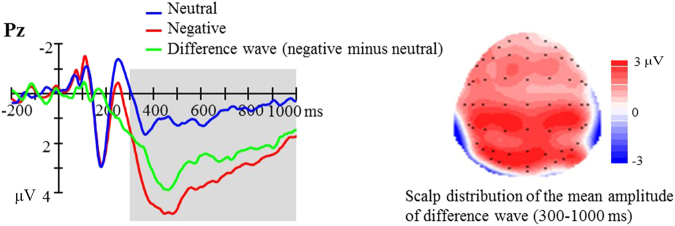
Left: ERPs time locked to the onset of neutral (blue line) and negative (red line) pictures as well as their difference wave (green line). Right: The scalp topography of LPP difference wave in the time window of 300–1000 ms.

**Figure 2:**
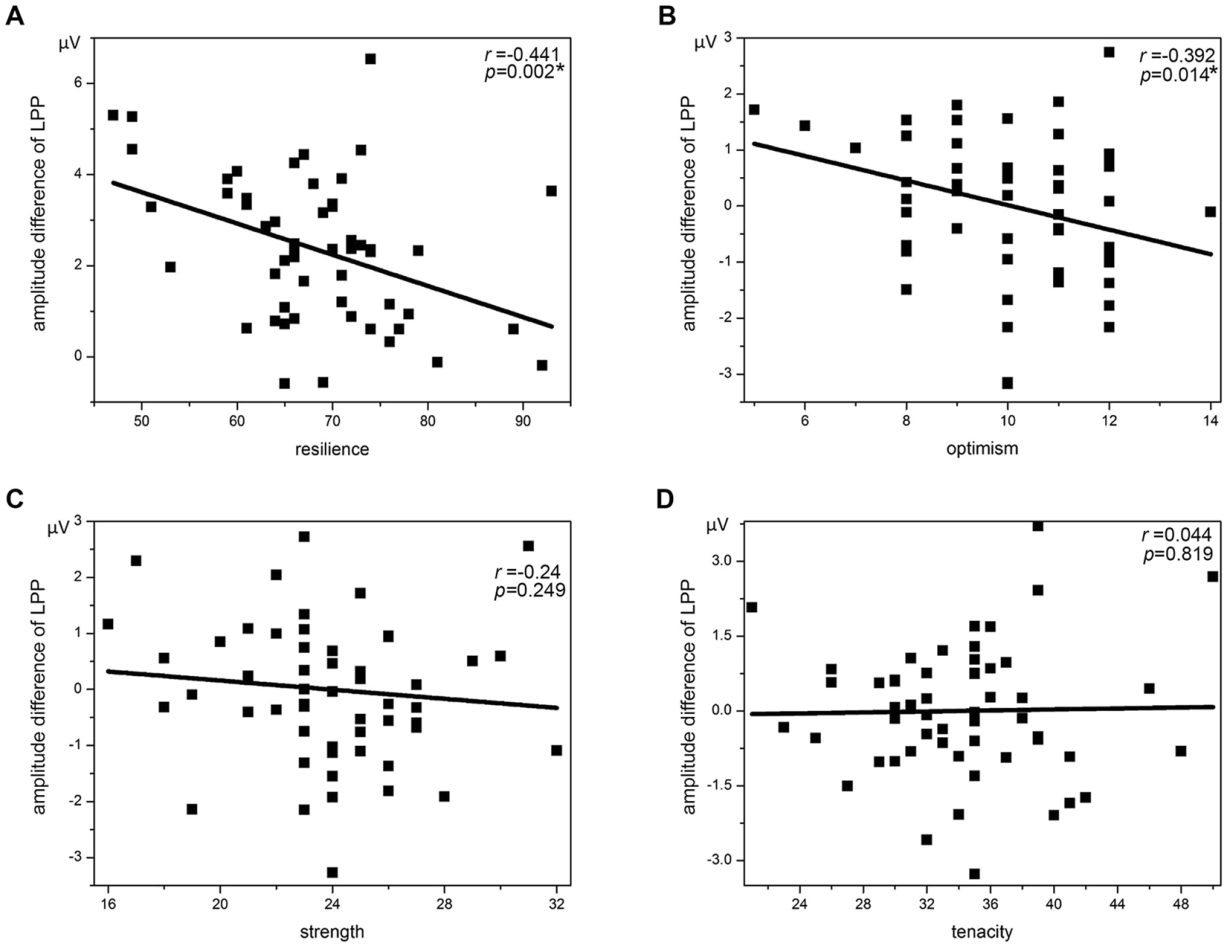
Linear association between the amplitude difference of LPP and different predictive variables of resilience (**A**), optimism (**B**), strength (**C**) and tenacity (**D**).

Figure 1 shows the ERPs time locked to the onset of neutral and negative pictures, as well as their difference wave at the representative electrode of Pz. Negative pictures elicited a larger LPP than neutral pictures. Used as the sole predictor, resilience did significantly explain variations of the difference scores of LPP (*β* = −0.441, *t* = −3.231, *p* = 0.002, *R*^2^ = 0.164). In addition, using the three composite variables as predictors, the regression analyses showed that the major predictive power came from optimism (*β* = −0.392, *t* = −2.553, *p* = 0.014, *R*^2^ = 0.262) while contributions from tenacity and strength were not significant (detailed statistics shown in Table 1). LPP associations with different predictive variables are graphically shown in Fig. 2.

**Table 1:** Regression results with combined and separate resilience factors.

Variables	*β*	SE of *β*	*t*	*p*	*R* ^2^
combined					
sex	−0.154	0.444	−1.147	0.257	0.001
age	0.046	0.204	−0.206	0.837	0.007
year of education	0.183	0.263	0.830	0.410	0.032
resilience	−0.441	0.023	−3.231	0.002*	0.164
separate					
sex	−0.107	0.428	−0.826	0.413	0.001
age	0.004	0.198	0.021	0.983	0.007
year of education	0.132	0.255	0.614	0.542	0.032
tenacity	0.044	0.054	0.23	0.819	0.076
strength	−0.24	0.101	−1.166	0.249	0.172
optimism	−0.392	0.139	−2.553	0.014^*^	0.262

*β*: regression coefficient; SE: standard error; *statistical significant.

## Discussion

The present study investigated whether resilience could predict negative emotional response in scalp ERP. The results showed larger amplitude of LPP responses to the negative compared to neutral targets, indicating an autonomic negative bias in attention allocation. This result is consistent with previous reports that negative information weighs more heavily in the brain^13^. In predicting this negative bias, our data has shown that resilience is negatively associated with this negative emotional response; and among the three composite factors of resilience, optimism is the major driving factor of this relationship.

The present findings have provided support for our main hypothesis of the predictive association between resilience and physiological brain responses to negative emotion. Presumably, behaviorally observed association between resilience and aversive coping could be partially mediated by this reduced neurophysiological responses. This scalp-recorded neurophysiological response may reflect brain mechanisms for emotion regulation involving both the dorsal executive network and the ventral affective network. For example, a simultaneous EEG and functional MRI (fMRI) study has shown that LPPs elicited by unpleasant pictures were associated with increased brain activation in the ventral lateral prefrontal cortex (PFC) and insula^16^. In contrast, deliberate emotion regulation with resilience is associated with increased brain activation in dorsal prefrontal cortices^17^. Therefore, resilient individuals may offset the negative emotion by increased activation in dorsal PFC that inhibits aversive responses in the ventral affective network. This inhibitive influence may also involve medial PFC input to brain stem and limbic structures that regulate stress control^18^. In addition to this effortful top-down emotion regulation, given the notion that a LPP could also originate from the locus coeruleus and release of norepinephrine^15^, resilience could also be associated with a relatively blunted autonomic alerting system extending from brainstem to a wide section of the cortex.

Considering the three composite factors of resilience, it was shown in the present results that optimism was the major contributor to the inhibiting neural response to negative emotion. In other words, resilience may regulate negative emotion specifically through optimism. Previous research has suggested that resilient individuals may use positive emotion to bounce back from negative emotional experiences^19^ and initiation of positive emotion could be facilitated by an optimistic trait^20^. Thus, mirroring individuals with high resilience, psychological treatment for affective/mood disorders could be improved by more involvements of positive reappraisal^21^. In fact, in addition to mood enhancement, optimism may also be an important factor for diminishing the negative impact of pain on cognition^22^; or even for intervention strategies targeting neural plasticity^23^. Additionally, besides LPP for detections of attentional bias towards negative emotion, the earlier ERP component of P2 could also be used in detections of a similar bias towards positive emotion^24,25^. Given the natural association between optimism and positive emotion, combined ERP indices jointly considering both emotionally positive and negative responses could be a more sensitive and specific biomarker of perceived emotion and regulation.

Negative emotional responses were induced by individual perceptions of the aversive pictures in the present study. However, with this negative emotion, the present paradigm did not require a behavioral output from the subjects. In other words, the present study only examined the effect of ongoing aversive perception but did not consider the effect that this negative emotion could impose on subsequent cognition and behavior. Since optimism measures one’s confidence in resisting ongoing adversity, while tenacity and strength underlie the sense of commitment and reintegration subsequent to setbacks^26,27^, it is possible that the lack of significant involvement of tenacity and strength could be due to the lack of a behavioral component subsequent to the induction of negative emotion. Particularly in the case of the aforementioned study examining pain induced dysregulation^22^, while increased optimism was reported to counteract pain-related deterioration on executive task performance, it could be of future research interest to examine whether this counteraction is stronger with increased tenacity or strength. Aside from the lack of a specific behavioral task, an additional limitation associated with the present paradigm of passive viewing was reduced task engagement. Without an explicit action requirement, subjects’ active engagement was difficult to be ascertained; thus, the increased variation across trials and participants may in turn hamper the ERP derivation and associated statistical power. With a more stringent control of task engagement and performance, future studies may capitalize on the increased sensitivity to further investigate associations between resilience and additional features of LPP, such as rise time to peak, recovery time, and duration of response^15^.

In conclusion, the present study confirmed a negative association between resilience and neurophysiological brain responses to negative emotion. Additionally, among the three composite factors of resilience, optimism was the crucial factor dominating this negative association. The present results complemented and extended previous behavioral studies^11^ with neurophysiological evidence, supporting the predictive value of resilience for identification of individuals sensitive to aversive challenge.

## Methods

### Participants

Fifty-four undergraduate students (22.57 ± 1.67 years, 35 males) participated in the present study. Incentives were provided to compensate for participants’ time spent in this study. They were recruited from college students enrolled in universities in Beijing. All participants provided informed consent. All subjects were right handed with normal or corrected-to-normal vision. All subjects had no history of head-injury or neurological disease. In addition, participants were free of major medical illnesses and were not taking medication. This study was approved by the Ethics Committee of Human Experimentation in the Institute of Psychology, Chinese Academy of Sciences. All methods used were in accordance with institutional guidelines and regulations.

### Stimuli

Thirty emotionally negative (valence: 2.48 ± 0.56, arousal: 5.66 ± 0.53) and thirty neutral (valence: 5.0 3 ± 0.34, arousal: 2.92 ± 0.49) pictures selected from the International Affective Picture System (IAPS)^28^ were used in the present study. These 60 pictures (IAPS IDs shown in the supplementary material) were repetitively presented in three blocks with each block exhausting all pictures in a random sequence.

### Questionnaire

Resilience was measured using the Chinese version of the Connor-Davidson Resilience Scale (CD-RISC)^5^, validated in Chinese samples^12^. This scale includes 25 items, and each item is rated on a five-point scale ranging from 0 (not true at all) to 4 (true nearly all the time). The CD-RISC was initially developed with five factors including tenacity, tolerance of negative effect, secure relationships, control, and spirituality^5^. However, given the cultural specificity, it was reorganized into three factors of tenacity, strength, and optimism for the Chinese population via exploratory factor analysis^12^. In this 3-factors CD-RISC, the tenacity describes an individual’s equanimity, promptness, perseverance, and sense of control under the situations of hardship and challenge. The strength focuses on individual’s capacity of recovering and becoming strong after setback and past experiences. The optimism measures one’s confidence in resisting adverse events. This modification was previously validated in a Chinese sample^12^ with a higher total score indicating a higher level of resilience.

### Experimental procedure

After completing questionnaires of the adapted CD-RISC, all subjects were settled in a quiet room. They were instructed to fixate on a cross in the center of the screen and then to passively watch sequential presentations of the aforementioned pictures. Negative and neutral pictures were randomly presented with a duration of 1000 ms and an inter-stimulus-interval varied between 1200 and 1800 ms. Upon completion of each block, subjects were asked to take a short rest for one or two minutes.

### EEG acquisition

EEG was recorded from 64 scalp sites using an elastic cap with Ag/AgCl electrodes mounted according to the international 10–20 system (Neuroscan Inc., Charlotte, North Carolina, USA). We adopted an on-line reference (for recording) to the left mastoid and an off-line (for analysis) algebraic reference to the average of the left and right mastoids. The vertical electrooculogram (VEOG) was recorded by a pair of electrodes placed above and below the left eye. The horizontal electrooculogram (HEOG) was recorded by a pair of electrodes placed 10 mm from the outer canthi of each eye. All electrode impedance was maintained below 5 kΩ. Signals were sampled at 1000 Hz with a 0.05–100 Hz band-pass filter.

### Data analysis

EEG data was processed using the software package of Scan 4.3 (Neuroscan, USA). Ocular artifacts were removed from the EEG signal using a regression algorithm implemented in the software^29^. Data was then digitally filtered with a 30 Hz low-pass filter and epoched into segments of 1200 ms (200 ms before stimulus onset) time-locked to picture onsets. Segments were rejected with an amplitude threshold of ±100 μV for artifact control. Subsequently, EEG amplitudes of each epoch were baseline corrected by subtracting the mean of pre-stimulus interval. We derived ERPs time locked to the stimulus onset for both the negative and neutral conditions. Difference waves between these two conditions were considered emotion-related responses. Based on the previous^30,31^ and the present observations that these emotion-related responses maximized at the electrode of Pz, the present analysis focused on the late positive portion of this difference wave at Pz in the time window of 300–1000 ms.

The CD-RISC measures resilience as a summary variable of three composite variables of tenacity, strength, and optimism. The present analysis was performed in two steps, examining (i) if resilience, as a summary variable, can predict emotional responses in LPP; and if yes, (ii) the respective contribution from the three composite variables. Therefore, a multivariate linear regression analyses (SPSS 22.0) was performed twice, with the independent regressor being one general measure of resilience, as well as being three composite measures of tenacity, strength, and optimism, respectively. In these multiple regression analyses, the difference scores of LPP from Pz was used as the dependent variable. Given the reported associations between resilience and gender^32^, age^32^, and year of education^33^, these demographic variables were included as covariates for confounding factor control. In the present sample, the resilience scores exhibited a significant gender difference (*r* = −0.311, *p* = 0.022), but insignificantly correlated with age (*r* = 0.08, *p* = 0.564) and years of education (*r* = −0.044, *p* = 0.75).

### Data availability

The datasets generated during and/or analysed during the current study are available from the corresponding author on reasonable request.

## Electronic supplementary material

Supplementary Information
