# Supplementary material for: Negative association between resilience and event-related potentials evoked by negative emotion
Source: Sci Rep. 2018 May 8;8:7149. doi: 10.1038/s41598-018-25555-w (PMC5940768; doi:10.1038/s41598-018-25555-w)
Supplement: Supplementary file 1 — Supplementary Information [file 41598_2018_25555_MOESM1_ESM.docx]

**Negative association between resilience and event-related potentials evoked by negative emotion**

Dan Chen^a, b^^,#^ , Jianhui Wu^a, b,#^, Zhuxi Yao^c,d,e^, Kaikai Lei^a, b^, Yuejia Luo^a, b*^, Zhihao Li^a, b,^*

^a^ School of Psychology and Sociology, Shenzhen University, Shenzhen, People’s Republic of China

^b^ Shenzhen Key Laboratory of Affective and Social Cognitive Science, Shenzhen University,

Shenzhen, Guangdong, People’s Republic of China

^c^ Key Laboratory of Behavioral Science, Institute of Psychology, Chinese Academy of Sciences, Beijing, China

^d^ Department of Psychology, University of Chinese Academy of Sciences, Beijing, China

^e^ Donders Institute for Brain, Cognition and Behavior, Department for Cognitive Neuroscience, Radboud University Medical Centre, Nijmegen, The Netherlands

**#**These authors contributed equally to this work

*Correspondence addressed to:

Zhihao Li, PhD. or Yuejia Luo, PhD.

School of Psychology and Sociology, Shenzhen University.

Science and Technology Building, 536,

3688 Nanhai Ave., Shenzhen 518060, Guangdong, People’s Republic of China.

E-mail: zhihao_li@szu.edu.cn, or luoyj@szu.edu.cn

Pictures’ number from IAPS:

Neutral pictures: 2214, 2215, 2372, 2381, 2383, 2440, 2480, 2495, 2514, 2516, 2580, 2749, 2850, 2870, 2880, 5520, 5530, 5740, 6150, 7004, 7006, 7031, 7034, 7060, 7090, 7185, 7187, 7205, 7234, 7950

Negative pictures: 1111, 1275, 2751, 3015, 3051, 3062, 3064, 3102, 3130, 3160, 3550, 6244, 6530, 6834, 9007, 9120, 9180, 9253, 9400, 9405, 9415, 9430, 9432, 9433, 9500, 9520, 9530, 9592, 9611, 9920
